# Supplementary material for: Thouless quantum walks in topological flat bands
Source: Light Sci Appl. 2026 May 20;15:244. doi: 10.1038/s41377-025-02140-1 (PMC13190768; doi:10.1038/s41377-025-02140-1)
Supplement: Supplementary file 1 — Supplemental material [file 41377_2025_2140_MOESM1_ESM.pdf]

Thouless quantum walks in topological flat bands
Supplementary information

Carlo Danieli,[1] Claudio Conti,[2,3] Laura Pilozzi,[1,3,*] and Valentina Brosco[1,2]

[1]Institute for Complex Systems, National Research Council (ISC-CNR), Via dei Taurini 19, 00185 Rome, Italy
[2]Department of Physics, University of Sapienza, Piazzale Aldo Moro 5, 00185 Rome, Italy
[3]Research Center Enrico Fermi, Via Panisperna 89a, 00184 Rome, Italy

PUMPING CYCLES $\mathcal{C}_{\mathcal{T}}^{++}$, $\mathcal{C}_{\mathcal{T}}^{-+}$, $\mathcal{C}_{\mathcal{T}}^{+-}$ AND $\mathcal{C}_{\mathcal{T}}^{--}$

In this section, we detail the actions of the pumping cycles $\mathcal{C}_{\mathcal{T}}^{\xi s}$ upon the symmetric and antisymmetric states $|p_n\rangle = \frac{|c_n\rangle+|d_n\rangle}{\sqrt{2}}$ and $|q_n\rangle = \frac{|c_n\rangle-|d_n\rangle}{\sqrt{2}}$. These cycles $\mathcal{C}_{\mathcal{T}}^{\xi s}$ are spherical triangles on the hyperplane $J_c = J_d$ for $s = +1$ and $J_c = -J_d$ for $s = -1$, and have anti-clockwise orientation for $\xi = +1$ and clockwise orientation for $\xi = -1$. Their corresponding holonomic transformations are

$$W_{\mathcal{C}_{\mathcal{T}}^{\xi s}} = e^{i\xi\frac{k}{2}(\sigma_0 - s\sigma_z)} \tag{1}$$

The four resulting cycles $\mathcal{C}_{\mathcal{T}}^{++}$, $\mathcal{C}_{\mathcal{T}}^{-+}$, $\mathcal{C}_{\mathcal{T}}^{+-}$ and $\mathcal{C}_{\mathcal{T}}^{--}$ are shown in Fig. S1(a,d,g,j) and they implement the following operators

$$\mathcal{C}_{\mathcal{T}}^{++} : \sum_n \Big[|p_{n+1}\rangle\langle p_n| + |q_n\rangle\langle q_n|\Big] \qquad \mathcal{C}_{\mathcal{T}}^{-+} : \sum_n \Big[|p_{n-1}\rangle\langle p_n| + |q_n\rangle\langle q_n|\Big] \tag{2}$$

$$\mathcal{C}_{\mathcal{T}}^{+-} : \sum_n \Big[|p_n\rangle\langle p_n| + |q_{n+1}\rangle\langle q_n|\Big] \qquad \mathcal{C}_{\mathcal{T}}^{--} : \sum_n \Big[|p_n\rangle\langle p_n| + |q_{n-1}\rangle\langle q_n|\Big] \tag{3}$$

as explicitly shown in Fig. S1

QUANTUM WALK EQUATIONS AND QUASI-ENERGIES

In this section, we derive Eq. (10) of the main text and the Floquet quasi-energies For quantum walks generated by cycles $\mathcal{C}_{\mathcal{T}}^{\xi+}\mathcal{C}_{\mathcal{R}}(\theta)$ the one time-step advancement from t to $t+1$ in vector form reads

$$\begin{pmatrix}\psi_n^p(t+1)\\ \psi_n^q(t+1)\end{pmatrix} = \begin{pmatrix}\cos\theta & \sin\theta\\ 0 & 0\end{pmatrix}\begin{pmatrix}\psi_{n-\xi}^p(t)\\ \psi_{n-\xi}^q(t)\end{pmatrix} + \begin{pmatrix}0 & 0\\ -\sin\theta & \cos\theta\end{pmatrix}\begin{pmatrix}\psi_n^p(t)\\ \psi_n^q(t)\end{pmatrix} \tag{4}$$

Subtract in both sides $\begin{pmatrix}\psi_n^p(t)\\ \psi_n^q(t)\end{pmatrix}$ and add and subtract in the right hand side $\begin{pmatrix}\cos\theta & \sin\theta\\ 0 & 0\end{pmatrix}\begin{pmatrix}\psi_n^p(t)\\ \psi_n^q(t)\end{pmatrix}$. By introducing the finite difference

$$\partial_t\begin{pmatrix}\psi_n^p(t)\\ \psi_n^q(t)\end{pmatrix} = \begin{pmatrix}\psi_n^p(t+1)\\ \psi_n^q(t+1)\end{pmatrix} - \begin{pmatrix}\psi_n^p(t)\\ \psi_n^q(t)\end{pmatrix} \qquad -\xi\partial_n\begin{pmatrix}\psi_n^p(t)\\ \psi_n^q(t)\end{pmatrix} = \begin{pmatrix}\psi_{n-\xi}^p(t)\\ \psi_{n-\xi}^q(t)\end{pmatrix} - \begin{pmatrix}\psi_n^p(t)\\ \psi_n^q(t)\end{pmatrix} \tag{5}$$

Eq. (4) reads

$$\partial_t\begin{pmatrix}\psi_n^p(t)\\ \psi_n^q(t)\end{pmatrix} = -\xi\begin{pmatrix}\cos\theta & \sin\theta\\ 0 & 0\end{pmatrix}\partial_n\begin{pmatrix}\psi_n^p(t)\\ \psi_n^q(t)\end{pmatrix} + \begin{pmatrix}\cos\theta & \sin\theta\\ -\sin\theta & \cos\theta\end{pmatrix}\begin{pmatrix}\psi_n^p(t)\\ \psi_n^q(t)\end{pmatrix} - \begin{pmatrix}\psi_n^p(t)\\ \psi_n^q(t)\end{pmatrix} \tag{6}$$

Via the Pauli matrixes σ_i and the identity matrix σ_0, Eq. (6) reads

$$\partial_t\begin{pmatrix}\psi_n^p(t)\\ \psi_n^q(t)\end{pmatrix} = -\xi\left[\cos\theta\frac{\sigma_0+\sigma_z}{2} + \sin\theta\frac{i\sigma_y+\sigma_x}{2}\right]\partial_n\begin{pmatrix}\psi_n^p(t)\\ \psi_n^q(t)\end{pmatrix} + [(\cos\theta-1)\mathbb{I} + \sin\theta i\sigma_y]\begin{pmatrix}\psi_n^p(t)\\ \psi_n^q(t)\end{pmatrix} \tag{7}$$

For quantum walks $\mathcal{C}_{\mathcal{R}}\mathcal{C}_{\mathcal{T}}^{\xi-}$, the one time-step advancement from t to $t+1$ in vector form reads

$$\begin{pmatrix}\psi_n^p(t+1)\\ \psi_n^q(t+1)\end{pmatrix} = \begin{pmatrix}0 & 0\\ -\sin\theta & \cos\theta\end{pmatrix}\begin{pmatrix}\psi_{n-\xi}^p(t)\\ \psi_{n-\xi}^q(t)\end{pmatrix} + \begin{pmatrix}\cos\theta & \sin\theta\\ 0 & 0\end{pmatrix}\begin{pmatrix}\psi_n^p(t)\\ \psi_n^q(t)\end{pmatrix} \tag{8}$$

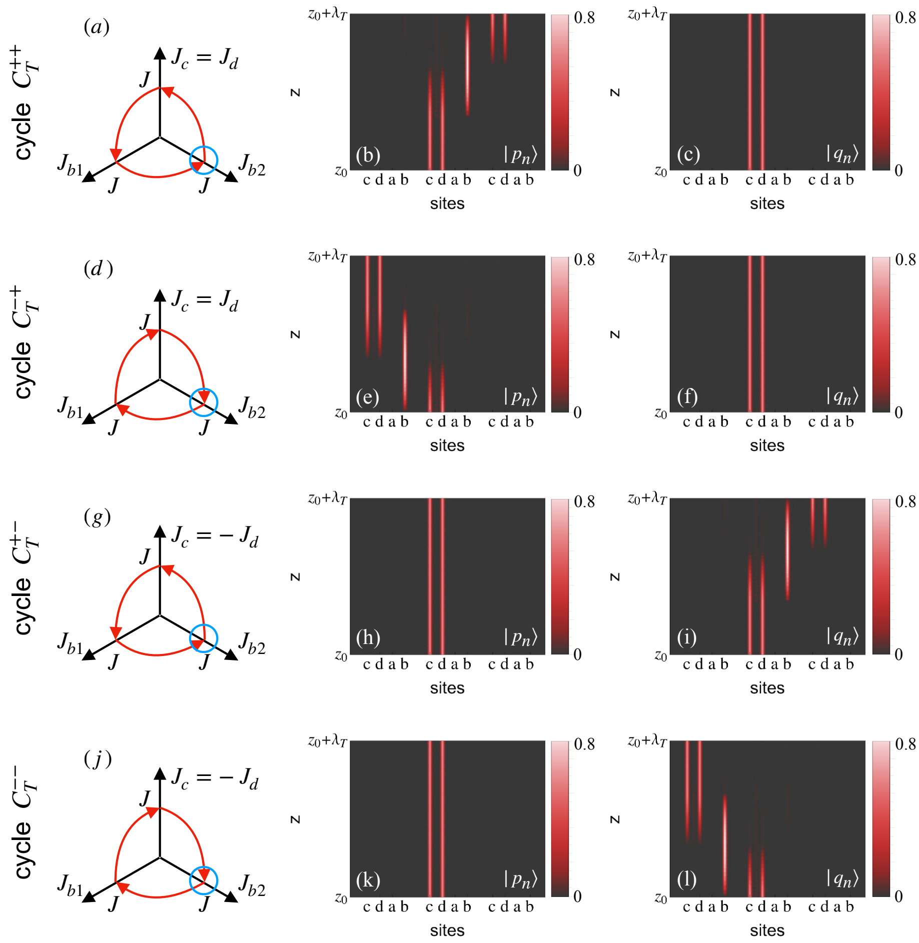

FIG. S1. (a) Sketch of cycle $\mathcal{C}_{\mathcal{T}}^{++}$. (b) and (c) Propagation of $|p_n\rangle$ and $|q_n\rangle$ respectively over one cycle period. The blue circles indicate the initial point. (d-f) Same as (a-c) for cycle $\mathcal{C}_{\mathcal{T}}^{-+}$. (g-i) Same as (a-c) for cycle $\mathcal{C}_{\mathcal{T}}^{+-}$. (j-l) Same as (a-c) for cycle $\mathcal{C}_{\mathcal{T}}^{--}$.

Alike in the previous case, subtracting in both sides $\begin{pmatrix}\psi_n^p(t)\\ \psi_n^q(t)\end{pmatrix}$ and add and subtract in the right hand side $\begin{pmatrix}0 & 0\\ -\sin\theta & \cos\theta\end{pmatrix}\begin{pmatrix}\psi_n^p(t)\\ \psi_n^q(t)\end{pmatrix}$, Eq. (8) reads

$$\partial_t \begin{pmatrix}\psi_n^p(t)\\ \psi_n^q(t)\end{pmatrix} = -\xi \begin{pmatrix}0 & 0\\ -\sin\theta & \cos\theta\end{pmatrix} \partial_n \begin{pmatrix}\psi_n^p(t)\\ \psi_n^q(t)\end{pmatrix} + \begin{pmatrix}\cos\theta & \sin\theta\\ -\sin\theta & \cos\theta\end{pmatrix}\begin{pmatrix}\psi_n^p(t)\\ \psi_n^q(t)\end{pmatrix} - \begin{pmatrix}\psi_n^p(t)\\ \psi_n^q(t)\end{pmatrix} \tag{9}$$

Via the Pauli matrixes σ_i and the identity matrix σ_0, Eq. (9) reads

$$\partial_t \begin{pmatrix}\psi_n^p(t)\\ \psi_n^q(t)\end{pmatrix} = -\xi \left[\cos\theta\frac{\sigma_0-\sigma_z}{2} + \sin\theta\frac{i\sigma_y-\sigma_x}{2}\right] \partial_n \begin{pmatrix}\psi_n^p(t)\\ \psi_n^q(t)\end{pmatrix} + \left[(\cos\theta-1)\mathbb{I} + \sin\theta i\sigma_y\right]\begin{pmatrix}\psi_n^p(t)\\ \psi_n^q(t)\end{pmatrix} \tag{10}$$

Hence, for quantum walks $\mathcal{C}_{\mathcal{R}}\mathcal{C}_{\mathcal{T}}^{\xi s}$ the one time-step advancement from t to $t+1$ in vector form reads

$$\partial_t \begin{pmatrix}\psi_n^p(t)\\ \psi_n^q(t)\end{pmatrix} = -\xi \left[\cos\theta\frac{\sigma_0+s\sigma_z}{2} + \sin\theta\frac{i\sigma_y+s\sigma_x}{2}\right] \partial_n \begin{pmatrix}\psi_n^p(t)\\ \psi_n^q(t)\end{pmatrix} + \left[(\cos\theta-1)\mathbb{I} + \sin\theta i\sigma_y\right]\begin{pmatrix}\psi_n^p(t)\\ \psi_n^q(t)\end{pmatrix} \tag{11}$$

In k-space $\tilde{\psi}_k(t) = \sum_n \psi_n(t)e^{ikn}$, the one time-step advancement Eqs. (4,8) relative to a generic oriented quantum walk $\mathcal{C}_{\mathcal{R}}\mathcal{C}_{\mathcal{T}}^{\xi s}$ reads

$$\tilde{\psi}_k(t+1) = M_k^{\xi s}\tilde{\psi}_k(t) \qquad M_k^{\xi s} = \begin{pmatrix} e^{\frac{i}{2}\xi(s+1)k}\cos\theta & e^{\frac{i}{2}\xi(s+1)k}\sin\theta \\ -e^{-\frac{i}{2}\xi(s-1)k}\sin\theta & e^{-\frac{i}{2}\xi(s-1)k}\cos\theta \end{pmatrix} \tag{12}$$

For either $s = +1$ and $s = -1$, their eigenvalues $\lambda^{1,2}_{k,\theta}$ are

$$\lambda^{1,2}_{k,\theta} = e^{i\xi\frac{k}{2}}\left(\cos\theta\cos\frac{k}{2} \pm i\sqrt{1-\cos^2\theta\cos^2\frac{k}{2}}\right) = e^{i\xi\frac{k}{2}}e^{\pm i\Omega_{k,\theta}} \tag{13}$$

which yield the relation for the quasi-energies $\lambda^{1,2}_{k,\theta} = e^{iE^{1,2}_{k,\theta}}$

$$\cos E^{1,2}_{k,\theta} = \cos\left[\frac{k}{2} \pm \Omega_{k,\theta}\right] \qquad \Leftrightarrow \qquad E^{1,2}_{k,\theta} = \xi\left[\frac{k}{2} \pm \Omega_{k,\theta}\right] = \xi\left[\frac{k}{2} \pm \arccos\left(\cos\theta\cos\frac{k}{2}\right)\right] \tag{14}$$

NON ABELIANITY

In this section, we show examples of propagations of ThQW obtained by permuting coin $\mathcal{C}_{\mathcal{R}}$ and shift operators $\mathcal{C}^{\xi s}_{\mathcal{T}}$. In our tests, we fix the an initial state $|\Psi(z_0)\rangle = \frac{1}{\sqrt{2}}|p_0\rangle + \frac{1}{\sqrt{2}}|q_0\rangle$.

In Fig. S2(a,b) we show the propagation of quantum walks obtained by the two possible compositions of the shift $\mathcal{C}^{\xi s}_{\mathcal{T}}$ and the coin $\mathcal{C}_{\mathcal{R}}$ with angle $\theta = \frac{\pi}{4}$ – namely $BA = \mathcal{C}^{++}_{\mathcal{T}}\mathcal{C}_{\mathcal{R}}(\frac{\pi}{4})$ [panel(a)] and $AB = \mathcal{C}_{\mathcal{R}}(\frac{\pi}{4})\mathcal{C}^{++}_{\mathcal{T}}$ [panel (b)] respectively. Fig. S2(c) shows the distributions at 100 steps. The two distributions occupy the same volume, and the final distributions change due to the effective change of initial state.

To obtain three non-commuting operators via the two pumping cycles, we define $A = \mathcal{C}_{\mathcal{R}}(\frac{\pi}{4})$, $B = \mathcal{C}^{+-}_{\mathcal{T}}$ and $C = \mathcal{C}^{++}_{\mathcal{T}}\mathcal{C}_{\mathcal{R}}(\frac{\pi}{6})$. In Fig. S2(c,d) we show the propagation of quantum walks obtained by the two non-equivalente compositions – namely $CBA = \mathcal{C}^{++}_{\mathcal{T}}\mathcal{C}_{\mathcal{R}}(\frac{\pi}{6})\mathcal{C}^{+-}_{\mathcal{T}}\mathcal{C}_{\mathcal{R}}(\frac{\pi}{4})$ [panel(d)] and $BCA = \mathcal{C}^{+-}_{\mathcal{T}}\mathcal{C}^{++}_{\mathcal{T}}\mathcal{C}_{\mathcal{R}}(\frac{\pi}{6})\mathcal{C}_{\mathcal{R}}(\frac{\pi}{4})$ [panel (e)]. Fig. S2(f) shows the distributions at 100 steps. The two distributions are drastically different.

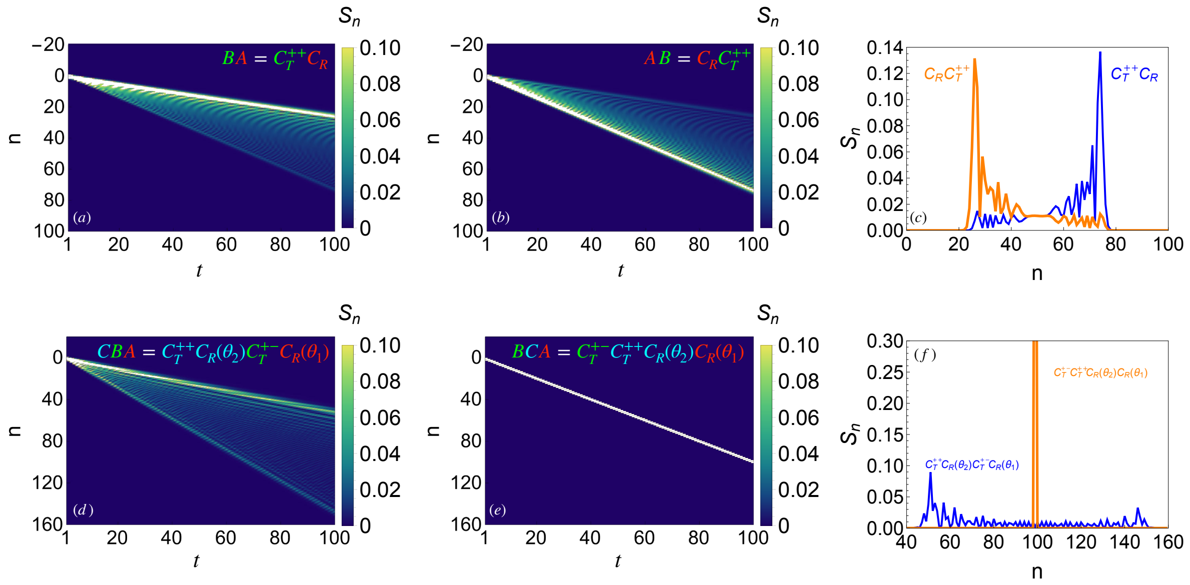

FIG. S2. (a,b) Propagation obtained via $BA = \mathcal{C}^{++}_{\mathcal{T}}\mathcal{C}_{\mathcal{R}}(\frac{\pi}{4})$ (a) and $AB = \mathcal{C}_{\mathcal{R}}(\frac{\pi}{4})\mathcal{C}^{++}_{\mathcal{T}}$ (b) for an initial state $|\Psi(z_0)\rangle = \frac{1}{\sqrt{2}}|p_0\rangle + \frac{1}{\sqrt{2}}|q_0\rangle$. and coin angle $\theta = \frac{\pi}{4}$. (c) Final distribution at $t = 100$ for $BA = \mathcal{C}^{++}_{\mathcal{T}}\mathcal{C}_{\mathcal{R}}(\frac{\pi}{4})$ (blue) and $AB = \mathcal{C}_{\mathcal{R}}(\frac{\pi}{4})\mathcal{C}^{++}_{\mathcal{T}}$ (orange). (d,e) Propagation obtained via $CBA = \mathcal{C}^{++}_{\mathcal{T}}\mathcal{C}_{\mathcal{R}}(\frac{\pi}{6})\mathcal{C}^{+-}_{\mathcal{T}}\mathcal{C}_{\mathcal{R}}(\frac{\pi}{4})$ (d) and $BCA = \mathcal{C}^{+-}_{\mathcal{T}}\mathcal{C}^{++}_{\mathcal{T}}\mathcal{C}_{\mathcal{R}}(\frac{\pi}{6})\mathcal{C}_{\mathcal{R}}(\frac{\pi}{4})$ (b) for an initial state $|\Psi(z_0)\rangle = \frac{1}{\sqrt{2}}|p_0\rangle + \frac{1}{\sqrt{2}}|q_0\rangle$. (f) Final distribution at $t = 100$ for $CBA = \mathcal{C}^{++}_{\mathcal{T}}\mathcal{C}_{\mathcal{R}}(\frac{\pi}{6})\mathcal{C}^{+-}_{\mathcal{T}}\mathcal{C}_{\mathcal{R}}(\frac{\pi}{4})$ (blue) and $BCA = \mathcal{C}^{+-}_{\mathcal{T}}\mathcal{C}^{++}_{\mathcal{T}}\mathcal{C}_{\mathcal{R}}(\frac{\pi}{6})\mathcal{C}_{\mathcal{R}}(\frac{\pi}{4})$ (orange).

* laura.pilozzi@cnr.it
